# Isolation and purification of recombinant immunoglobulin light chain variable domains from the periplasmic space of *Escherichia coli*

**DOI:** 10.1371/journal.pone.0206167

**Published:** 2018-10-22

**Authors:** Kieran Hand, Mark C. Wilkinson, Jillian Madine

**Affiliations:** Institute of Integrative Biology, University of Liverpool, Liverpool, United Kingdom; Massey University, NEW ZEALAND

## Abstract

Immunoglobulin light chain amyloidosis is the most common form of systemic amyloidosis. However, very little is known about the underlying mechanisms that initiate and modulate the associated protein aggregation and deposition. Model systems have been established to investigate these disease-associated processes. One of these systems comprises two 114 amino acid light-chain variable domains of the kappa 4 IgG family, SMA and LEN. Despite high sequence identity (93%), SMA is amyloidogenic *in vivo*, but LEN adopts a stable dimer, displaying amyloidogenic properties only under destabilising conditions *in vitro*. We present here a refined and reproducible periplasmic expression and purification protocol for SMA and LEN that improves on existing methods and provides high yields of pure protein (10-50mg/L), particularly suitable for structural studies that demand highly concentrated and purified proteins. We confirm that recombinant SMA and LEN proteins have structure and dimerization capabilities consistent with the native proteins and employ fluorescence to probe internalization and cellular localization within cardiomyocytes. We propose periplasmic expression and simplified chromatographic steps outlined here as an optimized method for production of these and other variable light chain domains to investigate the underlying mechanisms of light chain amyloidosis. We show that SMA and LEN can be internalised within cardiomyocytes and were observed to localise to the perinuclear area, assessed by confocal microscopy as a possible mechanism for underlying cytotoxicity and pathogenesis associated with amyloidosis.

## Introduction

To date, over 36 confirmed proteins are implicated in 50 known proteinopathies including Alzheimer’s, Parkinson’s, type 2 diabetes, Huntington’s and light chain associated diseases [1]. These debilitating disorders, collectively termed the “amyloidoses”, arise due to the misfolding of an otherwise native protein. Amyloid formation sees the loss of a protein's native state and adoption of aberrant conformations which result in the accumulation of insoluble fibrils that possess a highly ordered ultrastructure rich in β-sheet. The most common form of all systemic amyloidoses, which refers to the extracellular accumulation and deposition of the misfolded precursor protein at locations distant from the site of production, is Immunoglobulin (Ig) light chain (AL) amyloidosis with an incidence of 1 in every 100,000 [2]. In AL amyloidosis, patients with an underlying plasma cell dyscrasia show a significantly elevated population of intact or truncated Immunoglobulin light chains in circulation. A proportion of these light chains are able to adopt pathological conformations and accumulate as fibrillar aggregates causing irreversible damage to virtually all organs and tissues, leading to eventual death. The full-length light chain (consisting of components V_L_-J-C_L_) has been found to comprise the amyloid fibrils in a number of patients diagnosed with AL [3, 4], yet, mass spectrometric analysis of fat aspirates, has revealed that the variable domain, V_L_ has been the main fibrillar component for the majority (85%) of patients [5, 6]. This means that, each patient presents a light chain with a unique amino acid sequence, and consequently identifying the outcome of each mutation and identifying common themes that underlie aggregation in AL amyloidosis is a particularly challenging one.

As a model case we focus on the 114-amino acid immunoglobulin light chain variable domains from SMA and LEN that were originally isolated from patients suffering from either multiple myeloma or AL amyloidosis [7]. SMA was originally extracted post-mortem as amyloid fibrils from the lymph node of a patient suffering from AL amyloidosis [8] and displays significant aggregation propensity *in vitro*. LEN was isolated from urine as a Bence Jones protein of a patient diagnosed with multiple myeloma. No incidence of neuropathy or amyloid deposition was reported for LEN, despite significantly elevated levels of circulating light chain (50 g/L) in the urine [9]. *In vitro* characterization of these proteins revealed that SMA is significantly less stable than LEN [7, 10, 11], displaying enhanced fibrillation kinetics. Such dramatic differences arise only from a few amino acid substitutions, where SMA is altered by 8 residues (S29N, K30R, P40L, Q89H, T94H, Y96Q, S97T and I106L). *In vivo* the amyloid potential of these proteins as well as many other V_L_s and the effect on cells/mechanisms of toxicity has not been realised. A recombinant *E*. *coli* protein expression system was previously established for these V_L_ proteins, employing lysosyme cell disruption and purification using a multi-step chromatographic strategy of strong anion and cation exchange followed by size exclusion chromatography [7]. For LEN, yields were reported to be around 10 mg/L, while SMA was reported to be less. *Rognoni et al*., (2013) reported an optimized procedure for obtaining light chain proteins through recombinant expression and refolding from inclusion bodies [12]. However, we found that this method was not successful for SMA and LEN V_L_s resulting in a lower recovery from refolding, and elution with many co-contaminants. In addition to the problems with protein refolding noted above inclusion body aggregates are highly dynamic undergoing a continuous process of construction and deconstruction in which protein molecules in aggregated and soluble forms can freely exchange leading to highly structurally heterogeneous samples, which are undesirable for subsequent structural analysis [13, 14].

Previous groups have also demonstrated periplasmic expression for V_L_ expression using the *pel* B leader peptide for λ6 proteins [15, 16]. There is an increasing interest in recombinant expression of human immunoglobulin chain fragments for therapeutic use therefore improving expression yields and reproducibility of these systems are important factors to consider when designing expression strategies. Periplasmic expression offers a solution to many issues that arise when expressing proteins that are prone to mis-folding or aggregation. There are a range of different leader sequences that can and have been employed to direct light chain proteins and fragments to the periplasmic space e.g. amicyanin [17]. In the present work, we present a strategy exploiting periplasmic expression of V_L_s from the two light chain proteins SMA and LEN that improve on previous methods [7, 18]. Periplasmic expression can result in suboptimal yields and incomplete removal of peptide leader sequences [12]. We show that in our system we have improved yields, comparable with those obtained through the more complex refolding process and have complete removal of the leader sequence confirmed by mass spectrometry. We pay particular attention to this point, and also remove the presence of all solubility/ affinity tags to leave the protein free of any additional amino acids which may alter the stability of the protein; something undesirable when assessing the stability of these proteins with links to aggregation properties. We propose a simplified purification process of isoelectric precipitation followed by cation exchange chromatography for LEN, and with the addition of size exclusion chromatography for SMA, avoiding the use of lysozyme, which can cause complications in purification and confirm using multiple techniques that the proteins produced here have secondary structure consistent with other V_L_s. We then employ fluorescein isothiocyanate (FITC) labelled proteins to monitor internalisation and cellular localisation of SMA and LEN V_L_s into H9c2 rat cardiomyoblast cells using fluorescence microscopy. The ability of soluble light chains to internalize into cardiac fibroblasts, cardiomyocytes and renal cells and alter cellular metabolism and cellular ultrastructure has been documented previously [19–22]. Here, we investigate the ability of SMA and LEN V_L_s to internalize into cardiomyocytes and observe the sub-cellular localization as a potential mechanism for the initiation of cytotoxicity relevant to the pathological role of soluble immunoglobulin V_L_s in AL amyloidosis.

## Materials & methods

All chemicals were purchased from Sigma Aldrich, UK unless specified otherwise.

### Vectors and cloning

The LEN and SMA genes (Table 1) were synthetically produced by Life Technologies and inserted into pOPINO vector by the Oxford Protein Production Facility, UK. pOPINO comprises a signal sequence based on ompA prior to the protein of interest (POI), followed by a lysine residue and polyhistidine tag with ampicillin resistance. To remove the polyhistidine tag at the C-terminus, a premature stop codon was introduced directly upstream of the oligonucleotide containing lysine and 8 histidines by Site-directed, Ligase-Independent Mutagenesis (SLIM) [23] to generate the plasmid named LEN and SMApOPIN_ompAstop. All vectors were sequenced prior to use (Source Bioscience, Nottingham, UK).

**Table 1:** Biochemical properties of the two Ig V_L_ domains SMA and LEN. Amino acid sequence of the V_L_ domains were acquired from the Amyloid Light Chain Database (ALBase Boston university) using the patient ID’s as search. Amino acid differences between LEN and SMA (total of 8) are underlined. pI values and molecular weights were calculated using the ExPASy server [24].

Ig light chain	Molecular weight (Da)	pI	Amino acid sequence
**LEN**	12640.08	7.92	DIVMTQSPDSLAVSLGERATINCKSSQSVLYSSNSKNYLAWYQQKPGQPPKLLIYWASTRESGVPDRFSGSGSGTDFTLTISSLQAEDVAVYYCQQYYSTPYSFGQGTKLEIKR
**SMA**	12735.19	7.96	DIVMTQSPDSLAVSLGERATINCKSSQSVLYSSNNRNYLAWYQQKLGQPPKLLIYWASTRESGVPDRFSGSGSGTDFTLTISSLQAEDVAVYYCHQYYSHPQTFGQGTKLELKR

### Expression and purification

For protein expression, LB agar plates containing ampicillin (100 μg/mL) were streaked with *E*. *coli* C41 (DE3) cells transformed with either the LEN or SMA plasmid (pOPIN_ompAstop) and grown overnight at 37 ^o^C. A single colony was used to inoculate 50 mL of LB media supplemented with 100 μg/mL ampicillin and grown ~16 h at 37 ^o^C with agitation (200 rpm). This culture was used inoculate 1 L of LB media at a starting optical density (OD_600_) of 0.06–0.1. The culture was incubated at 30 ^o^C with shaking (110 rpm) until an OD_600_ ~ 0.75–0.85 was achieved. Protein expression was induced by the addition of 1 mM isopropyl β-D-1-thiogalactopyranoside (IPTG), and the culture incubated with shaking (110 rpm) for no longer than 16 h at 30 ^o^C for LEN, and 25 ^o^C for SMA (SMA was found to aggregate at higher temperatures). Cells were harvested by centrifugation (3360 x *g* for 10 min at 4 ^o^C).

#### Osmotic shock treatment

Osmotic shock was used to liberate the recombinant V_L_s from the periplasmic space of the host *E*.*coli* cells. Cell pellets were resuspended in a hypertonic osmotic shock solution (TES buffer) comprising 200 mM Tris, 5 mM EDTA, and 200 g/L w/v sucrose pH 8. Bacterial pellets were resuspended in 100 mL TES buffer (at 4 ^o^C) and incubated on ice for 30 min, with inversion at intervals of ~ 5 min to prevent sedimentation. Pellets were centrifuged again, at a higher centrifugal speed of 8000 x *g* for 10 min at 4 ^o^C, to sediment the pellet now in sucrose. It is important to note here that lower speed does not result in a firm pellet. The supernatant was discarded and the pellet rapidly resuspended in MilliQ water (35 mL/L of initial culture, 4 ^o^C) supplemented with one protease inhibitor tablet (cOmplete, Mini Protease Inhibitor Cocktail, ROCHE, UK) acting as a hypotonic solution. Solutions were again incubated for 30 min on ice before centrifugation at 23,000 x *g*, 30 min at 4 ^o^C to remove cellular debris. Periplasmic proteins were released from the periplasmic space in this final stage.

#### Dialysis and isoelectric precipitation

Hypertonic fractions containing LEN, or SMA were loaded into a 3500 Pierce Molecular weight cut-off (MWCO) pre-soaked dialysis membrane and the 35 mL dialysed against 3 L of 10 mM sodium acetate pH 5.0 at 4°C for ~ 36 h with 3 buffer changes. This resulted in the precipitation of a large proportion of host cell contaminants (assessed by SDS-PAGE) which were removed by centrifugation at 8000 x *g* for 15 min at 4°C. The supernatant containing the V_L_ of interest was used for further purification.

#### Purification

Supernatants were loaded directly onto 5 mL HiTrap SPFF columns (GE Healthcare, Sweden) mounted to an ÄKTA purifier chromatography system (GE Healthcare) at a flow rate of 0.75 mL per minute. A post load wash consisting of two column volumes (CV) of 10 mM acetate buffer, pH 5.0 was made before LEN was eluted using a 0–100 mM NaCl gradient over 20 mL. At this point, the purity of LEN fractions was deemed to be 95%, as judged by SDS-PAGE and reverse-phase high-performance liquid chromatography (RP-HPLC).

For SMA, which contained more contaminants than LEN, the protein was eluted with 5 CV of 10 mM Tris pH 8.0. For further purification of SMA, fractions were pooled, filtered using a 0.22-micron syringe filter to remove any aggregates and concentrated down to a volume of ~ 50 μL (from 4 L growth culture) using a 0.5 mL 10 kDa MWCO filter (Millipore, UK) prior to loading onto a HiLoad 16/60 Superdex 75 prep grade (GE Healthcare) size exclusion column pre-equilibrated with 20 mM Tris-HCl pH 7.5, 150 mM NaCl. Samples were injected through a 100 μL loop, flushed for 3 sample loop volumes and the chromatographic profile recorded at a flow rate of 1 mL/min. Proteins were eluted isocratically (1 CV) and the purity evaluated by SDS-PAGE and RP-HPLC.

Pure proteins (SMA and LEN) were concentrated and filtered into phosphate buffered saline (PBS) using 10 kDa MCWO filters. Samples were stored at > 3 mg/mL at 4°C where they showed no signs of degradation over a 12-month period (assessed by UV_280nm_, no visible precipitation, no degradation products on SDS-PAGE). Typical yields from 1 L of culture were ~ 10 mg for SMA and ~ 50 mg for LEN.

### Protein confirmation

The expression and purification of LEN and SMA were analysed by SDS-PAGE using 12% Tris-Tricine gels in a Bio-Rad gel electrophoresis system. Samples were solubilised in 4x Laemmli sample buffer for 5 min at 90 ^o^C prior to loading. Pierce unstained protein MW marker (Life technologies, UK) was loaded as molecular mass markers in all electrophoresis studies. Gels were run for 60 min at 165 V, stained with Coomassie Brilliant Blue G-250 0.25% (w/v) and destained with H_2_O, methanol, and acetic acid in a ratio of 45/45/10 (v/v/v).

The mass of the intact recombinant protein and the successful removal of the N-terminal ompA tag was confirmed by mass spectrometry. Protein samples were dialysed into 50 mM ammonium bicarbonate and the sample, at a protein concentration of 0.4 mg/mL, was infused into the nano electrospray source of the mass spectrometer [Waters Q-ToF Micro] via a gas tight syringe at a flow rate of 50 μL/hr. The positive ion mass spectrum of the sample was recorded in the m/z range 1,000–3,000. The recorded multiply charged mass spectrum was deconvoluted using the MaxEnt1 maximum entropy function in the MassLynx software (Waters, UK). The spectrum was processed over the mass range 5,000–25,000 Da.

RP-HPLC was also used to confirm purity: 10 μL samples were centrifuged at 10,000 *x g* for 5 minutes, and applied to a Phenomenex Aeris Widepore C4 column (150 x 2.1 mm) equilibrated in 0.08% Trifluoroacetic acid (TFA) attached to a Dionex ICS3000 HPLC system. Proteins were eluted with a linear gradient of 5–65% acetonitrile in 0.08% TFA: 0–40% over 60 min.

### Protein analysis

Circular dichroism (CD) was performed on a J1100 spectropolarimeter (JASCO, UK). CD spectra (250–180 nm) were acquired using a 0.2 mm cuvette, at 4 ^o^C using 10 μM proteins in 5 mM phosphate buffer, pH 7.5. Secondary structure content values were acquired using BeStSel [25].

The dimerisation ability of the recombinant proteins was characterised by Size Exclusion Chromatography—Multi-Angle Light Scattering (SEC-MALS). Purified SMA and LEN at concentrations of 1.0 mg/mL were applied directly to a HiLoad 16/60 Superdex 75 attached to an ÄKTA pure FPLC system equilibrated in 10 mM Tris-HCl pH 7.5, 150 mM NaCl. A DAWN 8+ and optilab T-rex Helios 8 (WYATT, UK) scattering detector was directed downstream flowpath of the SEC column. As a control lysozyme was run under identical conditions. Chromatograms were acquired at a flow rate of 0.75 mL/min at 25°C. Data was analysed using ASTRA v6.1 software.

### FITC labelling

In a similar method to the procedure of labelling light chains with Oregon green described previously [21], purified LEN and SMA were each dialysed into 10 mM PBS pH 7.4 overnight with multiple changes using a 3000 MWCO (Slide-A-Lyzer) Mini Dialysis unit (Thermo, UK). FITC labelling was performed according to manufacturer's guidelines with minor modifications to protein concentration (20 μM of each V_L_ protein used per reaction). Following incubation, samples were buffer exchanged into PBS using a NAP-5 column (GE Healthcare) to remove unbound FITC and Dimethyl sulfoxide present from the FITC storage solution. Buffer exchange was followed by diafiltration into 10 mM Tris pH 7.4 using a 10,000 MWCO centrifugal filter. Approximtely 10 cycles of dilution and concentration were performed until the dialysate appeared clear, and showed no absorbance at 495 nm (assessed by nanodrop), indicating there was no unbound FITC remaining. The amount of bound FITC was determined experimentally (nanodrop) according to the manufacturer's guidelines. Protein samples were only used when a FITC labelling ratio of 1 and above was achieved as this proved to be easily detectable under the microscope. CD and SEC-MALS were used to confirm that FITC labelling did not alter the secondary structure or dimerization capabilities of the proteins (data not shown).

### Protein internalisation

H9c2 rat cardiomyocytes (ATCC) were kindly gifted from Dr Parveen Sharma. Cells were cultured in Dulbecco’s Modified Eagle’s Medium supplemented with 10% fetal bovine serum and 5% penicillin. At the desired confluency (70–80%), cells were rinsed twice with PBS, subcultivated by trypsinisation and seeded into 24-well plates (Corning, Costar, The Netherlands) containing a 12 mm (diameter) 0.16–0.19 mm (depth) glass coverslip at a density of 40,000 cells/well (500 μL per well). Cells were left to adhere for 16 hours prior to experimentation.

Recombinant V_L_ proteins were added at a cell confluency judged to be below ~ 50% by microscopy to prevent overgrowth. Varying concentrations (as indicated in results section) of recombinant SMA and LEN were added in PBS and plates were imaged after 24 h. Negative controls (PBS alone) that were free of FITC labelled V_L_ domains were also performed.

#### Slide preparation

For detection of internalised V_L_s, cells were washed in permeabilisation buffer (1x PBS/0.2 Triton X -100 for 3 x 15 min), washed with PBS (10 min, room temperature) and incubated in PBS containing Hoechst stain (1:5000) and Phalloidin (1:250) for 20 min at room temperature. A final wash in PBS (2 x 10 min, room temperature) was conducted prior to mounting. For internalisation assays using FITC labelled proteins, following incubation with V_L_s cells were fixed (4% PFA, 30 min, room temperature) the reaction quenched (33 mM glycine in PBS 10 min, room temperature) and washed in PBS (2 x 15 min, room temperature).

Cells were mounted in the presence of ProLong Antifade (Thermo, UK), sealed using lacquer, stored at 4 ^o^C and visualised within 48 h of fixing to prevent fade and maximise signal intensity. At all possible stages, cells were kept in the dark to avoid light exposure. Control slides containing cells that were free of V_L_s were included for all assays and used to match exposure levels on the 488 nm channel.

#### Confocal microscopy

Internalisation experiments were imaged using an Axio observer z1 microscope (Zeiss, UK) equipped with ApoTome and a 40x Plan-Neoflaur oil immersion objective (Zeiss). Wavelengths of 488 nm, 568 nm and 680 nm were used to visualise FITC/Alexafluor conjugated antibody, Phalloidin and Hoechst respectively. For Z-stack, ~20 images were taken at 0.28 μm intervals. Analysis of Z-slice and 3D reconstruction of images was performed using Zeiss Zen blue v2.3. Exposure levels were kept consistent between experiments to allow for direct comparison with control slides.

## Results and discussion

### Vector generation

Here, we employ periplasmic expression of our POI using the ompA leader peptide (MKKTAIAIAVALAGFATVAQA) fused to the amino termini of each individual V_L_, where the protein is targeted by the SEC translocase pathway to the oxidising compartment of the periplasmic space. It is here where the ompA signal sequence is cleaved by a signal peptidase which leaves an unmodified amino terminus. This strategy has several attractions; the prokaryotic periplasm contains lower quantities of endogenous proteases and contaminating bacterial proteins [26] which negates the use of many initial purification steps. This compartment also contains the foldases; disulfide oxidoreductase (DsbA) and disulfide isomerase (DsbC) that are localised to the periplasmic space [27] and assist in correct folding and disulfide bond formation. We employed the pOPINO plasmid from OPPF, which encompasses an inducible T7 promoter, ampicillin resistance cassette and an N-terminal ompA leader sequence allowing for diffusion of the protein into the periplasmic space. However, we found that the His tag was preventing the POI being translocated efficiently into the periplasmic space therefore a premature “taa” stop codon was introduced directly upstream of the his-fusion using SLIM mutagenesis to generate pOPIN_ompAstop.

### Recombinant expression of V_L_ LEN

**Fig 1:**
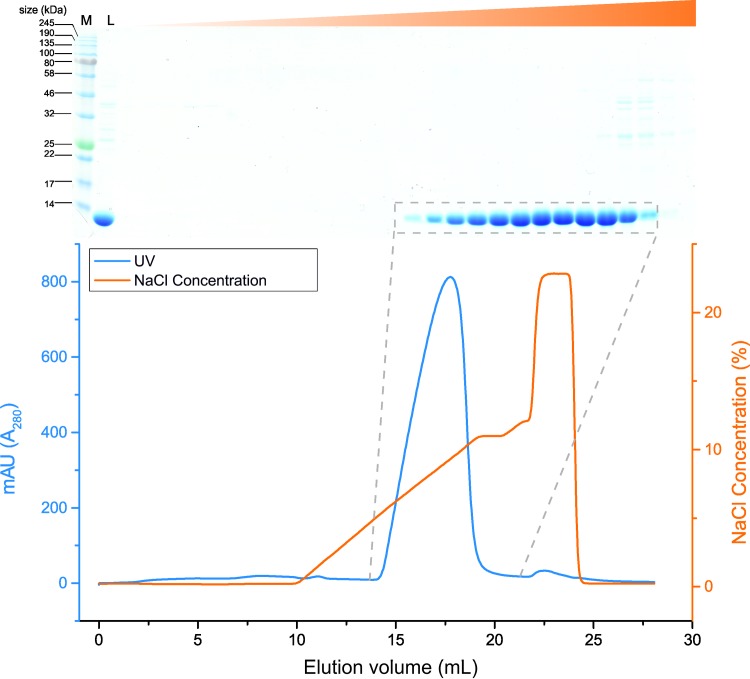
Representative elution profile and SDS-PAGE analysis of the purification of LEN by ion exchange chromatography using a gradient elution. LEN in sodium acetate pH 5.0 was applied to a 5 mL HiTrap SP HP column at a flow rate of 0.75 mL/min. A post load wash of 2 CV sodium acetate pH 5.0 was followed by the elution of LEN using a gradient of 0–100 mM NaCl. The purity of fractions (1 mL) corresponding to the elution peak were assessed by SDS-PAGE. Lane “L” shows load fraction, the remaining lanes are fractions 1–30 fractions that correspond to the elution profile. Target proteins were isolated as a single band which corresponds to the UV trace as shown (blue).

The modified plasmid was used to transform *E*.*coli* C41 (DE3) cells and protein was expressed with IPTG induction. Osmotic shock using sucrose was used to liberate proteins from the periplasmic space and SDS-PAGE analysis showed detectable levels of soluble protein expression, with a monomeric band of approximately ~13 kDa corresponding to the calculated theoretical molecular weights of LEN and SMA (Table 1)(S1A and S1B Fig). Osmotic shock is used to isolate proteins from the periplasm as a gentler method than other more traditional lysis techniques to leave the cytoplasmic space intact [28, 29]. It has been shown to act like a filtration step releasing small proteins less than 100 kDa [30], in turn reducing the number of contaminating proteins. The liberated protein mixture was then dialysed into 10 mM sodium acetate buffer pH 5.0 to remove sucrose and lower the pH. This resulted in the precipitation of a large amount of host cell contaminants (S1C Fig). Following removal of these precipitated contaminants, cation exchange chromatography was used to purify recombinant protein to high levels of homogeneity with a single-step process. A linear salt gradient in the mobile phase eluted LEN as a single peak (Fig 1) whilst host organism contaminants remained bound.

### Recombinant expression of V_L_ SMA

**Fig 2:**
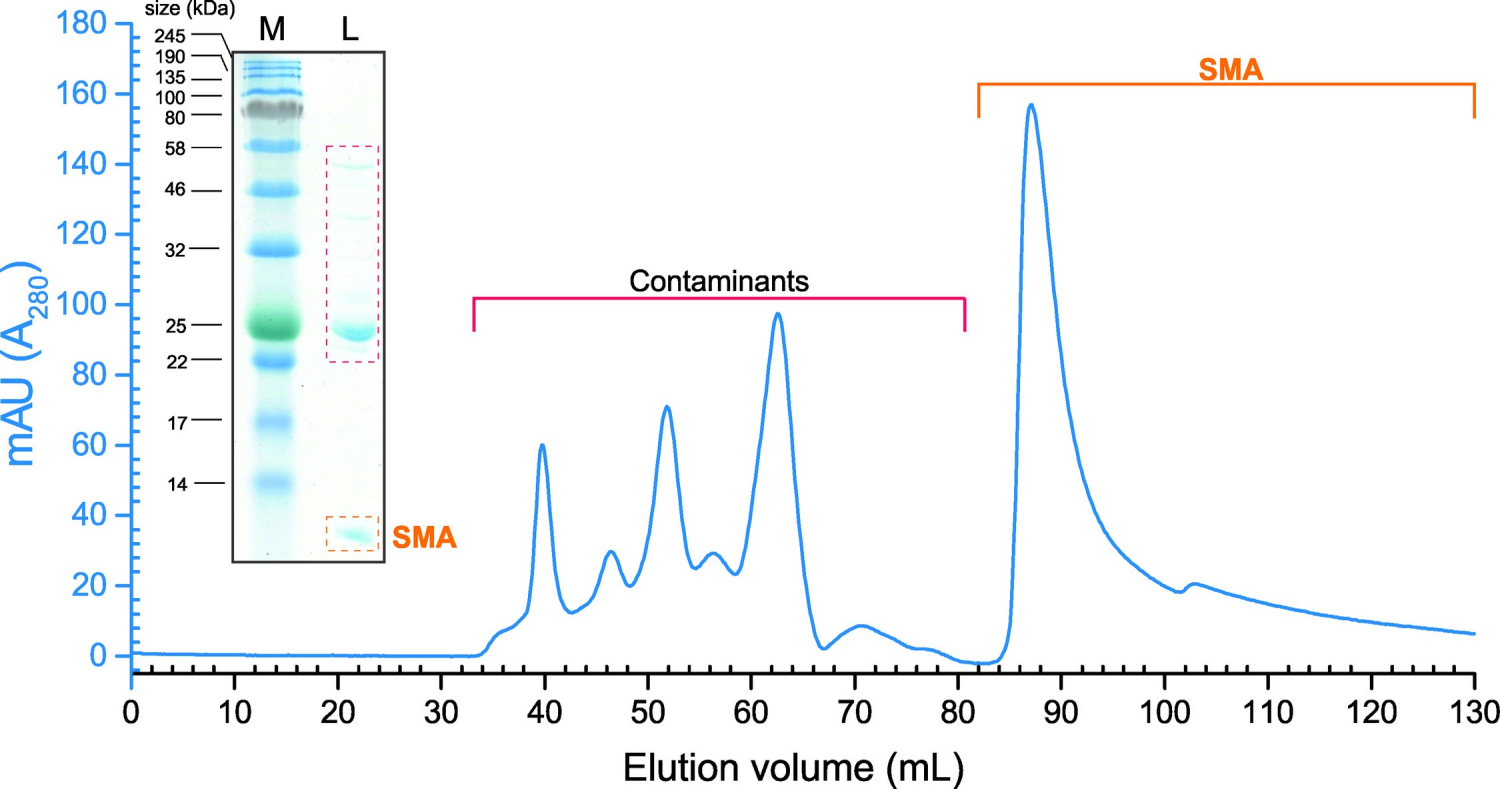
Final purification strategy for SMA isolated from C41 cells. SDS-PAGE analysis of SMA following ion exchange chromatography revealed higher molecular weight contaminants. SEC chromatography was used as a polishing step to separate the recombinant protein from host cell contaminants. Protein fractions were dialysed into 10 mM Tris-HCl pH 7.5, 150 mM NaCl, passed through a 0.22 micron filter, then concentrated before application to a prepacked Superdex 16/60 Superdex 75 column at 1 ml/min. As shown, the protein elutes at ~ 82 ml and displays the typical asymmetric peak characteristic of the V_L_s which reflects the simultaneous dissociation and re-binding of the protein on the column.

**Fig 3:**
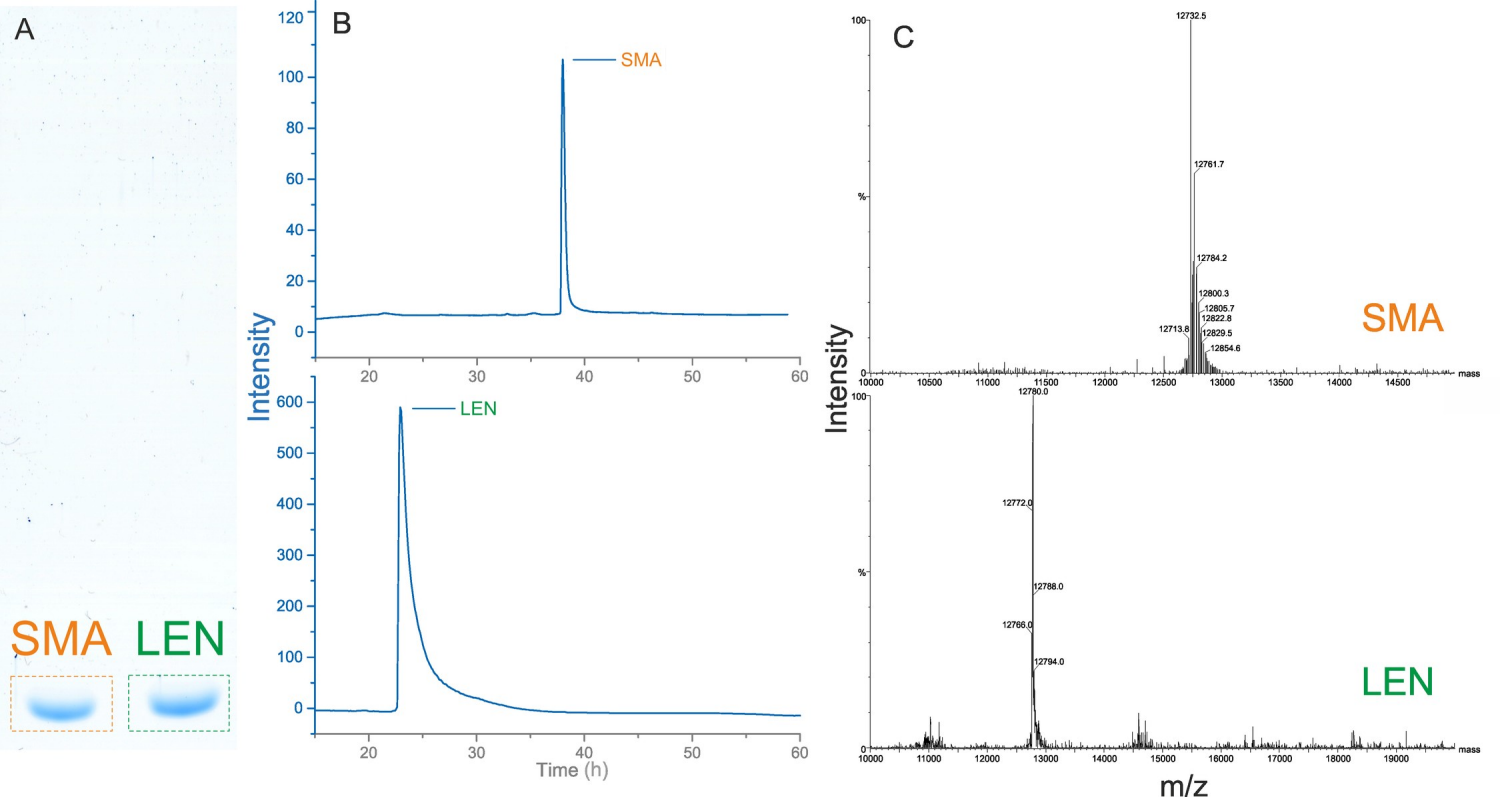
Purity analysis for LEN and SMA. (A) The degree of purity of SMA and LEN was assessed by SDS-PAGE analysis and deemed >95% pure, where the proteins can be seen migrating as a single band just below the 14 kDa band of the molecular marker (as can be seen in Figs 1 and 2). (B) The molecular ion peaks with the highest intensities correspond to the theoretical molecular weight of LEN (m/z 12640) and monomeric SMA (12735). Both spectra confirm that the ompA leader sequence has been successfully removed following translocation into the periplasmic space. Predictions were performed using peptidemass [32]. (C) RP-HPLC profile of purified LEN and SMA. UV absorption was measured at 280 nm. Purity for each protein was estimated to be >98%. The differences in elution time are due to column availability.

Expression in C41 cells for 16 h following IPTG induction produced soluble SMA in the periplasmic space liberated by osmotic shock as described for LEN above. SMA required expression at a lower temperature than LEN to prevent aggregation. In addition, expression exceeding 16 h resulted in the formation of SMA in inclusion bodies or SDS-resistant oligomers (experimentally verified by western blot). As for LEN, isoelectric precipitation removed contaminating proteins prior to cation exchange chromatography. SMA was eluted from the SP column as described for LEN above, analysis of all fractions by SDS-PAGE confirmed the presence of SMA and host organism contaminants in the eluates, thus requiring further purification steps (Fig 2, inset). Prior to size exclusion chromatography, fractions containing SMA were again dialysed into sodium acetate pH 5.0 which resulted in further precipitation of bacterial contaminants before being applied to a size exclusion column. The protein elutes at ~ 82 ml and displays the typical asymmetric peak characteristic of the VLs which reflects the simultaneous dissociation and re-binding of the protein on the column [7, 31]. Fig 2 shows the successful separation of the contaminants to leave pure SMA (Fig 3A).

### Confirmation of recombinant Immunoglobulin light chain identity

The end purity degree of LEN and SMA containing fractions following all chromatographic procedures were assessed by SDS-PAGE and RP-HPLC (Fig 3), where the chromatograms reveal a single peak for each protein indicating high levels of purity and no other visible contaminants (Fig 3B). The isolated proteins were confirmed to be correct by mass spectrometric analysis with the highest intensity peak corresponding to the theoretical molecular weight of LEN (m/z 12640) and monomeric SMA (12735) (Fig 3C).

### Analysis of protein secondary structure

**Fig 4:**
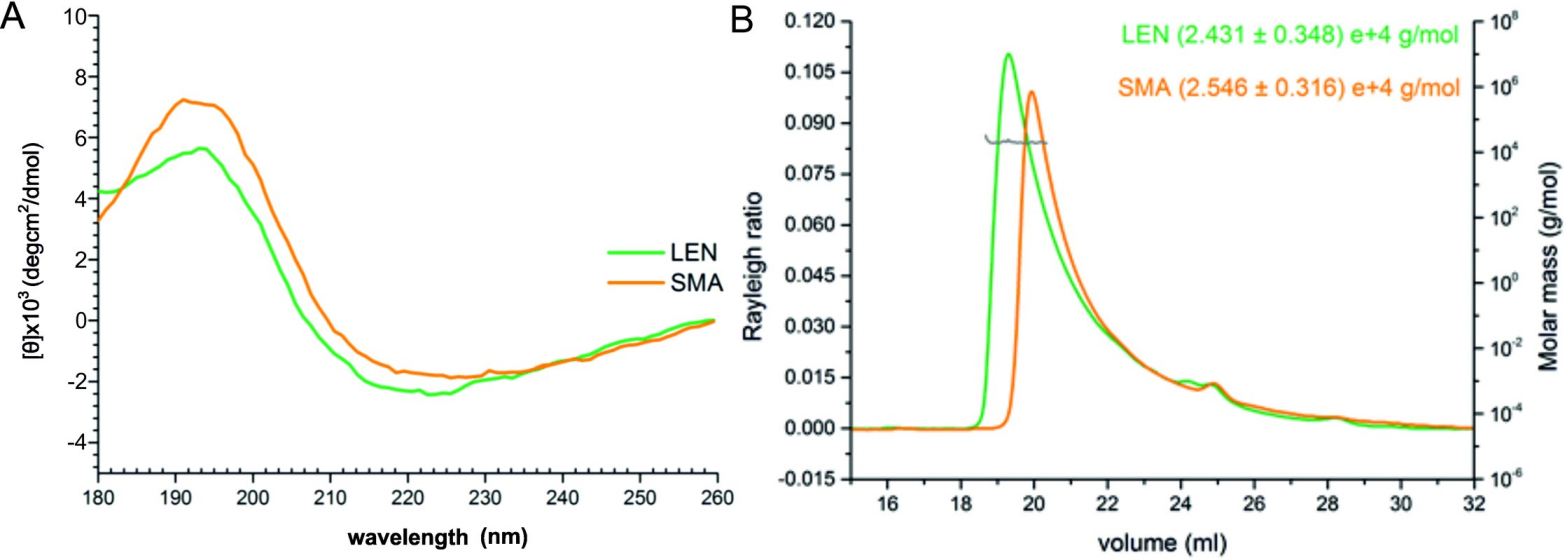
Analysis of fold and dimerisation state of LEN and SMA. (A) Far UV-CD spectra of V_L_ confirms correctly folded recombinant protein, displaying native β-sheet structure with minimum around 220 nm. Units are presented as mean residue ellipticity. CD experiments were performed with 20 μM protein in 5 mM sodium phosphate (pH 7.5) at 4°C. (B) SEC-MALS chromatogram confirming the dimerisation state of LEN and SMA. Both V_L_s elute as single asymmetrical peaks at a volume of ~18.5 mL and ~19.5 mL on a HiLoad 16/60 Superdex 75 size exclusion column showing that a single species is present. Concentration of each protein was 1 mg/mL.

Far UV-CD spectra of SMA and LEN confirm correctly folded recombinant protein, displaying native β-sheet structure with minimum around 220 nm (Fig 4A). BEST-SEL analysis of CD spectra indicates the protein to possess an Ig like fold through its fold recognition software [25]. Particularly evident for SMA is an elongated negative peak covering the region ~216–236 nm which incorporates the two areas of negative peaks previously observed for SMA [33], and other V_L_s [34, 35] attributed to β-sheet structure and arrangement of aromatic residues respectively [36]. V_L_s typically exist as a homodimer in low ionic strength buffer. To experimentally assess the ability of recombinant LEN and SMA to form dimers, their oligomerisation state was assessed by SEC-MALS (Fig 4B). The unique shape of the peak is consistent with other V_L_s characterised by size exclusion based methods [37–39]. Based on the calculated molecular weight of 24.31 kDa (LEN) and 25.46 kDa (SMA) both proteins are homodimers, corresponding to the theoretical molecular weights of dimers, confirming that both proteins are capable of dimerisation.

### Detection of internalised FITC-labelled VLs

**Fig 5:**
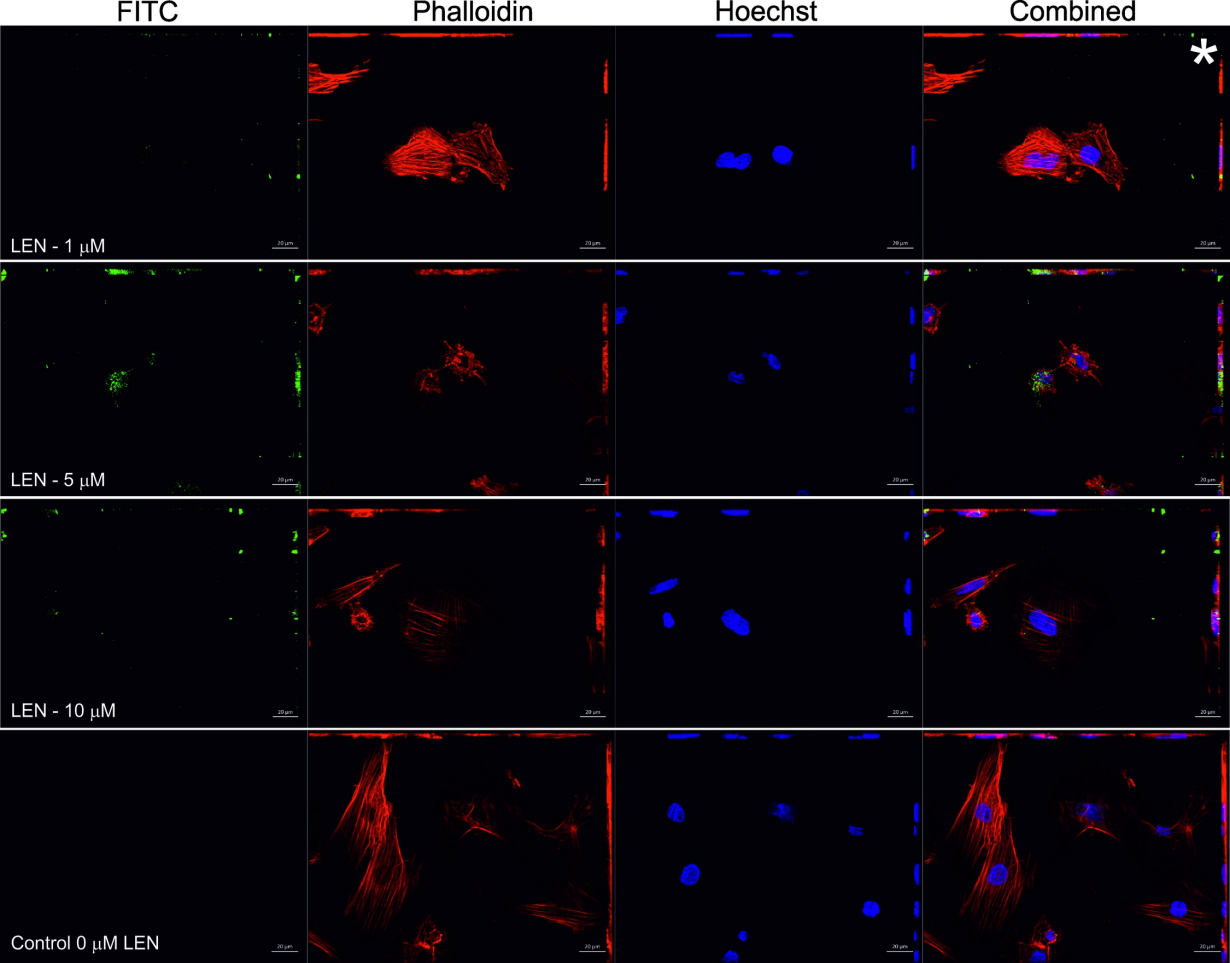
Internalisation of FITC conjugated LEN monitored by fluorescence microscopy. In three independent experiments, FITC labelled LEN (green) at concentrations of 1, 5 and 10 μM (indicated) were incubated with rat H9c2 cardiomyocytes for 24 h. Detection of the FITC signal was made by maximum intensity projections and Z-stack analysis (S3 Fig). A control experiment that was free of LEN was conducted. Combined shows all channels (blue Hoechst—nuclei, red Phalloidin—F-actin and green–FITC labelled LEN). Scale bar is 20 μM. Asterisk indicates image that was taken for further processing (see Fig 7).

H9c2 cardiomyocytes were incubated with FITC labelled protein at 1, 5, and 10 μM (final concentrations) for 24 h. The K_d_ of LEN is 10 μM [40] and SMA 40 μM [41], therefore both proteins are a combination of monomer and dimer at the concentrations used in these experiments. For an accurate comparison between samples incubated with or without FITC labelled protein, all images were taken using identical exposure levels. FITC conjugated LEN appears to be internalised in all experiments, with no visible differences between the different concentrations used (Fig 5). The distribution of LEN appears to be sparse, consistent with the analysis of AL-09 in HL-1 cardiomyocytes [21]. One striking observation is seen in H9c2 cells that were incubated with LEN at 5 μM, where strong staining is observed on a cell that appears to undergo apoptotic staging with altered cytoskeleton observed by phalloidin staining (Fig 5). Surrounding cells (past the field of view for this image) did not display any green signal. Cell viability analysis in the presence of SMA and LEN did not indicate any effect on cell viability at this concentration (S2 Fig and S1 Method). At this magnification, overall cell morphology appears largely unaffected by the addition of LEN_,_ where stained F-actin (red- Phalloidin) has a similar structure to control slides with the exception of the aforementioned 5 μM LEN slide (Fig 5).

**Fig 6:**
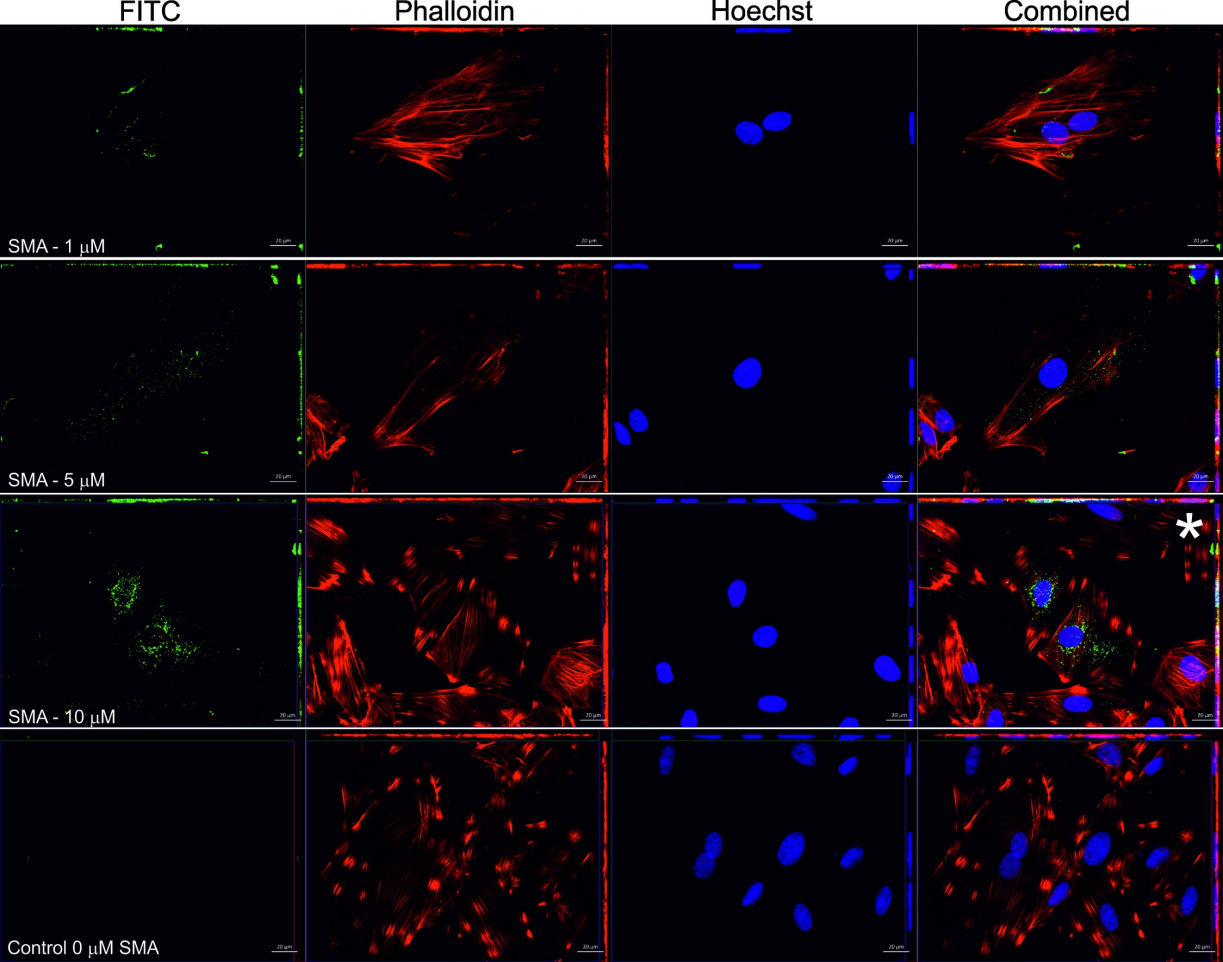
Internalisation of FITC conjugated SMA monitored by fluorescence microscopy. In multiple independent experiments, FITC conjugated SMA (green) at concentrations of 1, 5 and 10 μM (indicated) were incubated with rat H9c2 cardiomyocytes for 24 h. Detection of the FITC signal was made by maximum intensity projections and Z-stack analysis (see S4 Fig). A control experiment that was free of SMA was conducted. Combined shows all channels (blue Hoechst—nuclei, red Phalloidin—F-actin and green–FITC labelled LEN). Scale bar is 20 μM. Asterisk (*) in SMA 10 μM indicates data that was taken for further analysis (see Fig 8).

As was the case with LEN, FITC labelled SMA is detectable in all experiments (Fig 6). However, the level of internalisation for SMA at a concentration of 10 μM is most noteworthy. Despite this level of internalisation, the overall morphology appears unaffected where stained F-actin (Phalloidin, red) retains the striated pattern in both control and variable domain incubated slides similar to cells incubated with LEN.

### Internalised FITC-LEN and SMA: Z-stack and 3D reconstruction

**Fig 7:**
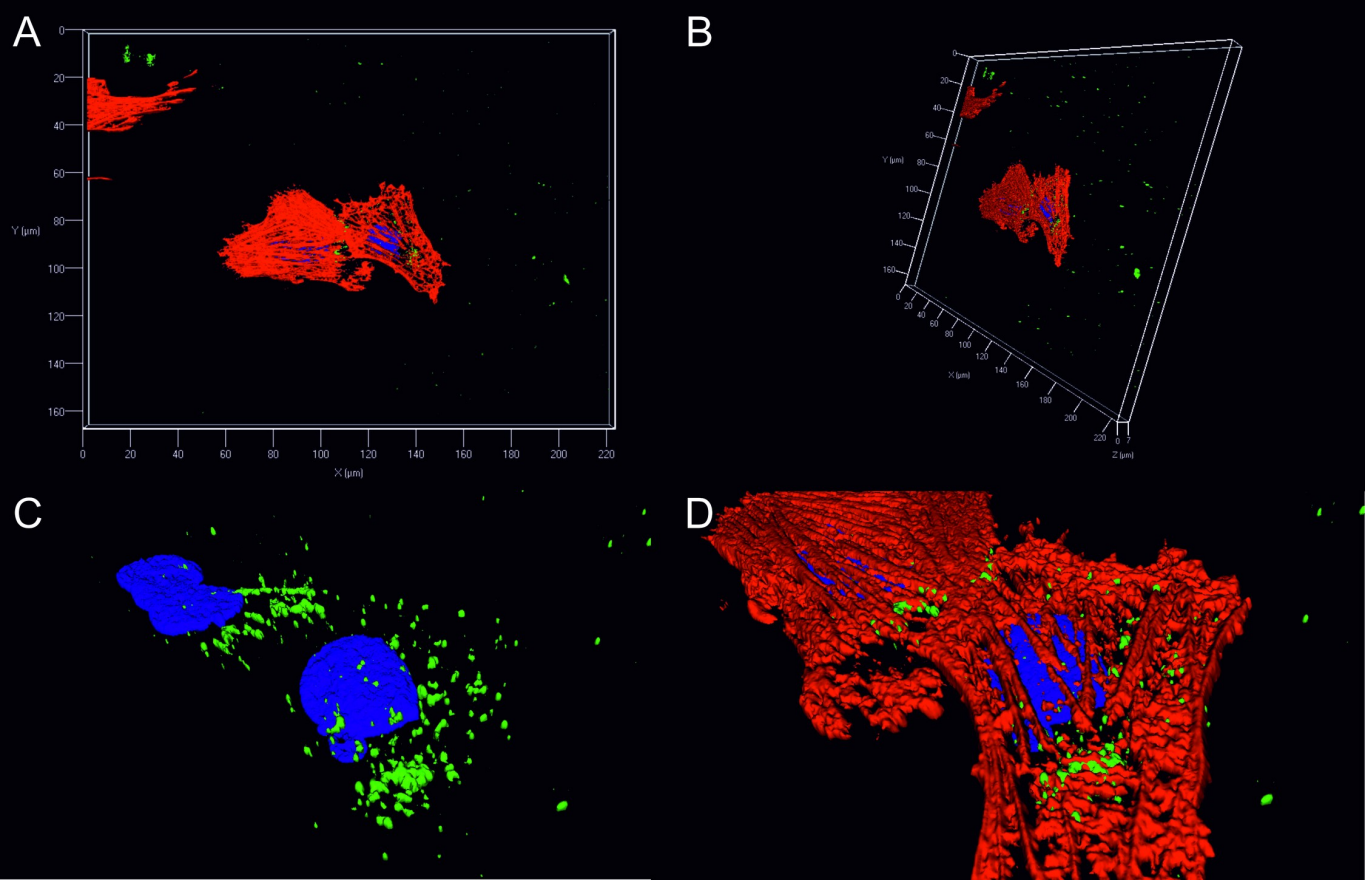
Internalisation and localisation of FITC conjugated LEN assessed by 3D reconstruction. 1 μM FITC labelled LEN (green) that was previously incubated with rat H9c2 cardiomyocytes for 24 h was taken for further analysis in order to determine the location of the signal. Combined shows all channels (blue Hoechst—nuclei, red Phalloidin—F-actin and green–FITC labelled LEN). A and B show 3D reconstruction of three colour z-stacked image of internalisation of FITC conjugated LEN rotated on x and y-axis. C and D show zoomed-in image without (C) and with (D) Phalloidin channel engaged (red) to demonstrate that the FITC signal is not surface bound.

Next, we wished to determine the subcellular origin of the FITC signals. Here, optical sections of the experiment using 1 μM FITC labelled LEN (marked asterisk Fig 5) were analysed further. As illustrated in the two-colour z-stack image (S3 Fig), the most intense FITC (green) signal is found on the focal planes (12, 13 and 14) that are occupied by the cell nucleus (Hoechst–blue) suggesting that the V_L_ is not surface bound, and the observed signal originates from inside the cell. Although extracellular fluorescent material is present, the majority of green signal is intracellular (illustrated in z-slice 13, S3 Fig). To complement this analysis, we again used the 1 μM LEN 2D confocal image shown in Fig 6, but here performed three-dimensional reconstruction of the entire z-stack. A complete 3D construction that covers the entire depth of the cell (Fig 7) illustrates that the FITC signal is not surface bound. This is most clear in panels C and D where FITC signal can be seen surrounding the cell nuclei.

**Fig 8:**
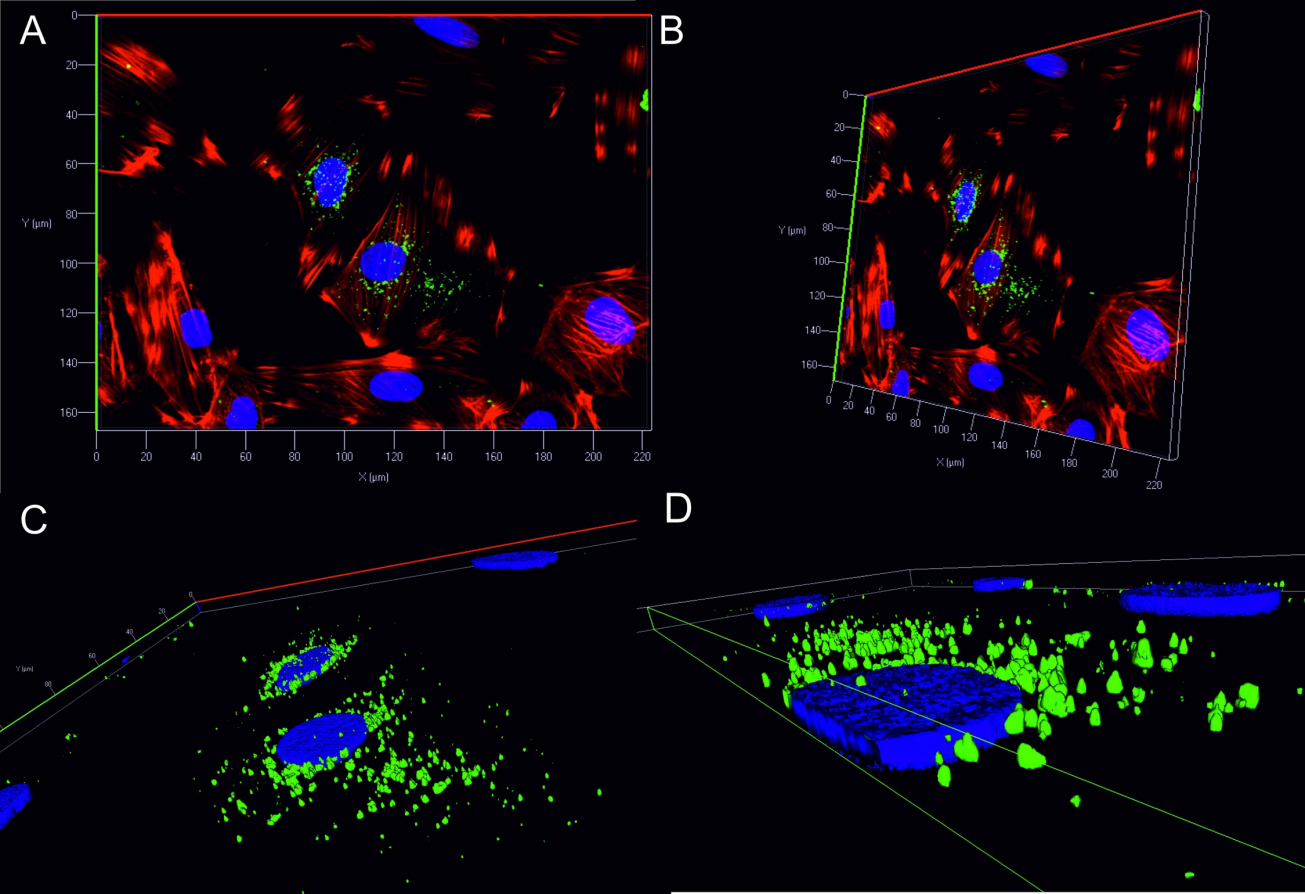
Internalisation and localisation of FITC conjugated SMA assessed by 3D reconstruction. 10 μM FITC labelled SMA (green) that was previously incubated with rat H9c2 cardiomyocytes for 24 h was taken for further analysis in order to decipher the location of the signal. Combined shows all channels (blue Hoechst—nuclei, red Phalloidin—F-actin and green–FITC labelled SMA). A and B show 3D reconstruction of three colour z-stacked image of internalisation of FITC conjugated SMA rotated on x and y-axis. C and D show zoomed-in images with to demonstrate that the FITC signal is not surface bound.

Similarly, the origin of the FITC-SMA signal was also investigated further and z-stack images of cardiomyocyte cells incubated with 10 μM SMA (previous Fig 6) were analysed by maximum intensity projection (S4 Fig). The z-slice containing the most intense FITC-signal is shown in z-13 (marked with asterisk on S4 Fig, and shown in top right panel zoomed-in) and clearly indicates the localisation of the fluorescent signal to be within the cell and not surface bound, where individual confocal sections z1 and z20 indicate the outside of the cell, and top of the cell respectively. For a clearer depiction to the origin of the FITC signal, z-stacked images underwent 3D reconstruction (Fig 8). Orthogonal cross section of the z-projection (panel C) clearly indicates the protein is localised to the perinuclear region of the cell and is not bound to the surface. Interestingly, it appears that some of the FITC conjugated SMA (green) is associated with the nucleus (Fig 8C) where FITC signal can be seen to originate within the nuclear space. While the ability of light chains to localise to perinuclear compartments have been noted [22], to the best of our knowledge a demonstration of intranuclear localisation of light chain variable domain has not previously been documented by such methods presented here. Similar staining patterns have however, been documented previously for other fluorescently tagged proteins and small molecules [42, 43].

## Summary & discussion

Comparisons between amyloidogenic and non-amyloidogenic V_L_s have demonstrated that variations in the amino acid sequence can lead to enhanced thermodynamically instability and a loss in structural integrity, relating to variations in the aggregation propensity of these proteins. Deciphering the outcome of these mutations in a structural—stability linked approach for every mutation is no trivial task, and routinely employs the use of site directed mutagenesis, equilibrium unfolding and refolding experiments [10, 44], analytical ultracentrifugation in combination with fibrillation based assays and high-resolution techniques such as X-ray crystallography [45] and more recently Nuclear Magnetic Resonance [10, 46–48]. It is here where recombinant protein expression through *E*.*coli* based methods has long been the established workhorse for providing an inexpensive, and easy manipulate and easy to culture source of protein to supply techniques that are demanding of high protein concentrations. Complications in protein expression can arise, particularly when attempting to study the outcome of a suspected destabilising mutation in V_L_ as this can lead to the formation of insoluble inclusion bodies that require many difficult steps to acquire soluble, pure protein suitable for analysis or can even be toxic to the bacterial host [49]. Indeed recombinant V_L_s studied to date are often isolated from inclusion bodies. Identifying ideal conditions that permit refolding of an intact protein let alone a fragment of an antibody into native topology can be a timely process. Such limitations and difficulties likely contribute to why the outcome of a particular mutation have been studied experimentally for only a handful of V_L_s despite there being thousands of different light chain sequences [50].

We present here an optimized periplasmic expression method, with purification employing a controlled osmotic shock procedure free of lysozyme or other chemical lysis methods that disrupts only the periplasmic space leaving the cytoplasmic space undisturbed. Furthermore, the addition of an isoelectric precipitation step dramatically reduces the level of contaminating host cell proteins reducing the number of chromatographic steps required for enhanced-scale production and improving purity and yield. Purity was deemed > 95% as confirmed by SDS-PAGE and RP-HPLC with protein sequences, and full cleavage of the ompA leader sequence confirmed using mass spectrometry. Estimated protein yields were ~ 50 mg/L for LEN and ~ 10 mg/L for SMA with little variation between preparations. This is a significant improvement on previous work for these proteins where yields were reported to be around 10 mg/L for LEN, and less for SMA using lysosyme cell disruption and multi-step chromatographic purification [7], or refolding from inclusion bodies [12]. In addition, we perform secondary structure analysis by CD spectroscopy, and use SEC-MALLS to confirm the ability of recombinant products to dimerise. We believe the detailed methods described here will be applicable to other V_L_ domains that may not be amenable to refolding techniques previously proposed [12] to produce high quality protein in sufficient quantities for functional and structural analysis when a protein source is no longer available from the AL diagnosed patient. We show that these recombinant sources of protein are suitable for fluorescent detection of κIV variable domains by conjugating highly pure fractions of SMA and LEN with FITC. Both proteins were internalized within cells and observed to localise to the perinuclear area, as assessed by z-stack confocal microscopy.

## Supporting information

S1 Method Cell Toxicity. (DOCX)

S1 Fig SDS-PAGE analysis of the expression and isolated of LEN and SMA. Both V_L_s were expressed and isolated from the periplasmic space of the host cell using osmotic shock. The success of the procedure was assessed by SDS-PAGE (Panels A and B). The gel lanes are marked: Lane M Pierce Unstained Protein MW Marker; Lane 1- Uninduced total bacterial proteins; Lane 2 –IPTG Induced total bacterial protein extract; Lane 3—the hypertonic solution. The target proteins LEN and SMA are indicated (dashed box). (C) A number of host cell contaminants were then removed using an isoelectric precipitation step.
(TIF)

S2 Fig The effect of SMA and LEN on rat cardiomyocyte toxicity. SMA and LEN (1, 5, and 10 μM as shown) were incubated with H9c2 cells for 24 h before analysis by CCK-8 assay, absorbance at 450 nm. Results are expressed as mean ± s.e.m following conversion to % viability. ANOVA with Dunnett's post-hoc analysis was performed (*p<0.05), n = 6 for live and dead controls and n = 3 for LC incubated cells.
(TIF)

S3 Fig Subcellular localisation of internalised FITC conjugated LEN assessed by analysis of Z-stack. Optical sectioning of complete z-stacks reveals FITC-labelled LEN (green) is on the same focal plane as the cell nucleus (Hoechst–blue) indicating the V_L_ is inside the cells and not surface bound. Top right panel shows enlarged image of z-slice 13 marked asterisks.
(TIF)

S4 Fig Subcellular localisation of internalised FITC conjugated SMA assessed by analysis of Z-stack. Optical sectioning of complete Z-stacks reveals FITC-labelled SMA (green) is on the same focal plane as the nuclei (Hoechst–blue) indicating the V_L_ is inside the cells and not surface bound. Top right panel shows enlarged image of z-slice 13 marked asterisks.
(TIF)
