# Supplementary material for: Isolation and purification of recombinant immunoglobulin light chain variable domains from the periplasmic space of Escherichia coli
Source: PLoS One. 2018 Oct 22;13(10):e0206167. doi: 10.1371/journal.pone.0206167 (PMC6197867; doi:10.1371/journal.pone.0206167)
Supplement: S1 Method — (DOCX) [file pone.0206167.s001.docx]

**Cell toxicity**

The toxicity of SMA and LEN in cell culture was assessed using cell counting kit-8 (CCK-8). Rat H9c2 cardiomyocytes (ATCC) were seeded into a 96-well plate at a density of 8,000 cells per well in a total of 80 μL Dulbecco’s Modified Eagle’s media that was free of phenol red. After 24 h, cells were treated with SMA or LEN at concentrations 1, 5 and 10 μM for an additional 24 h. Control live cells were cultured in media substituted with 20 % V_L_ storage buffer (to match protein samples, 10 mM Tris pH 7.4), and control dead cells contained 1 % Triton X-100. Final culture volumes totalled 100 μL. Following a further 24 h incubation, CCK-8 solution was added at 10 μL per well and incubated at 37 ^o^C for 2 hours before the measuring the absorbance at 450 nm. Wells containing V_L_s were performed in triplicate. For live and dead cell controls, experiments were repeated six times. Results are expressed as the mean ± s.e.m following conversion to % viability. A one-way analysis of variance (ANOVA) in conjunction with Dunnett's post-hoc analysis was carried out using PRISM (v7), and statistically significant results indicated (*p<0.05).
