# Supplementary figures and images for: Isolation and purification of recombinant immunoglobulin light chain variable domains from the periplasmic space of Escherichia coli
Source: PLoS One. 2018 Oct 22;13(10):e0206167. doi: 10.1371/journal.pone.0206167 (PMC6197867; doi:10.1371/journal.pone.0206167)

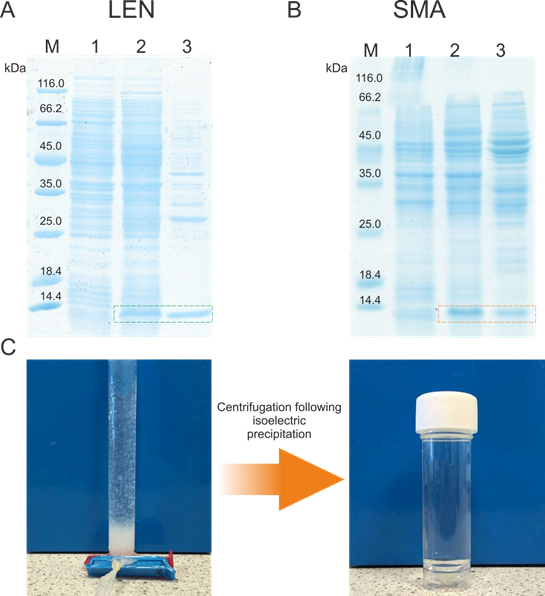

Supplement: S1 Fig — Both VLs were expressed and isolated from the periplasmic space of the host cell using osmotic shock. The success of the procedure was assessed by SDS-PAGE (Panels A and B). The gel lanes are marked: Lane M Pierce Unstained Protein MW Marker; Lane 1- Uninduced total bacterial proteins; Lane 2 –IPTG Induced total bacterial protein extract; Lane 3—the hypertonic solution. The target proteins LEN and SMA are indicated (dashed box). (C) A number of host cell contaminants were then removed using an isoelectric precipitation step. (TIF) [file pone.0206167.s002.tif]

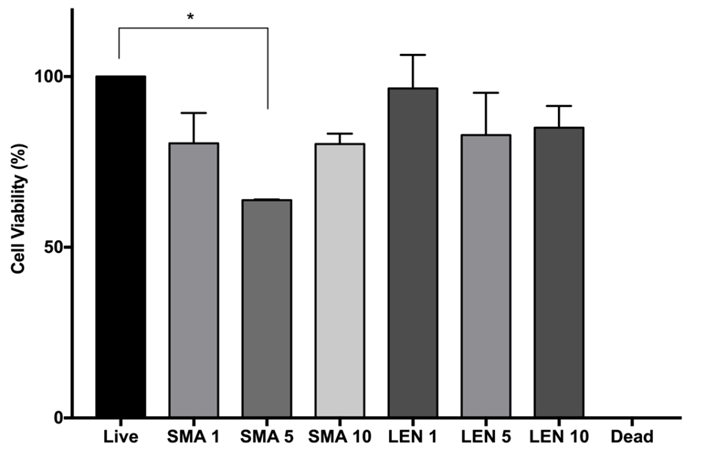

Supplement: S2 Fig — SMA and LEN (1, 5, and 10 μM as shown) were incubated with H9c2 cells for 24 h before analysis by CCK-8 assay, absorbance at 450 nm. Results are expressed as mean ± s.e.m following conversion to % viability. ANOVA with Dunnett's post-hoc analysis was performed (*p<0.05), n = 6 for live and dead controls and n = 3 for LC incubated cells. (TIF) [file pone.0206167.s003.tif]

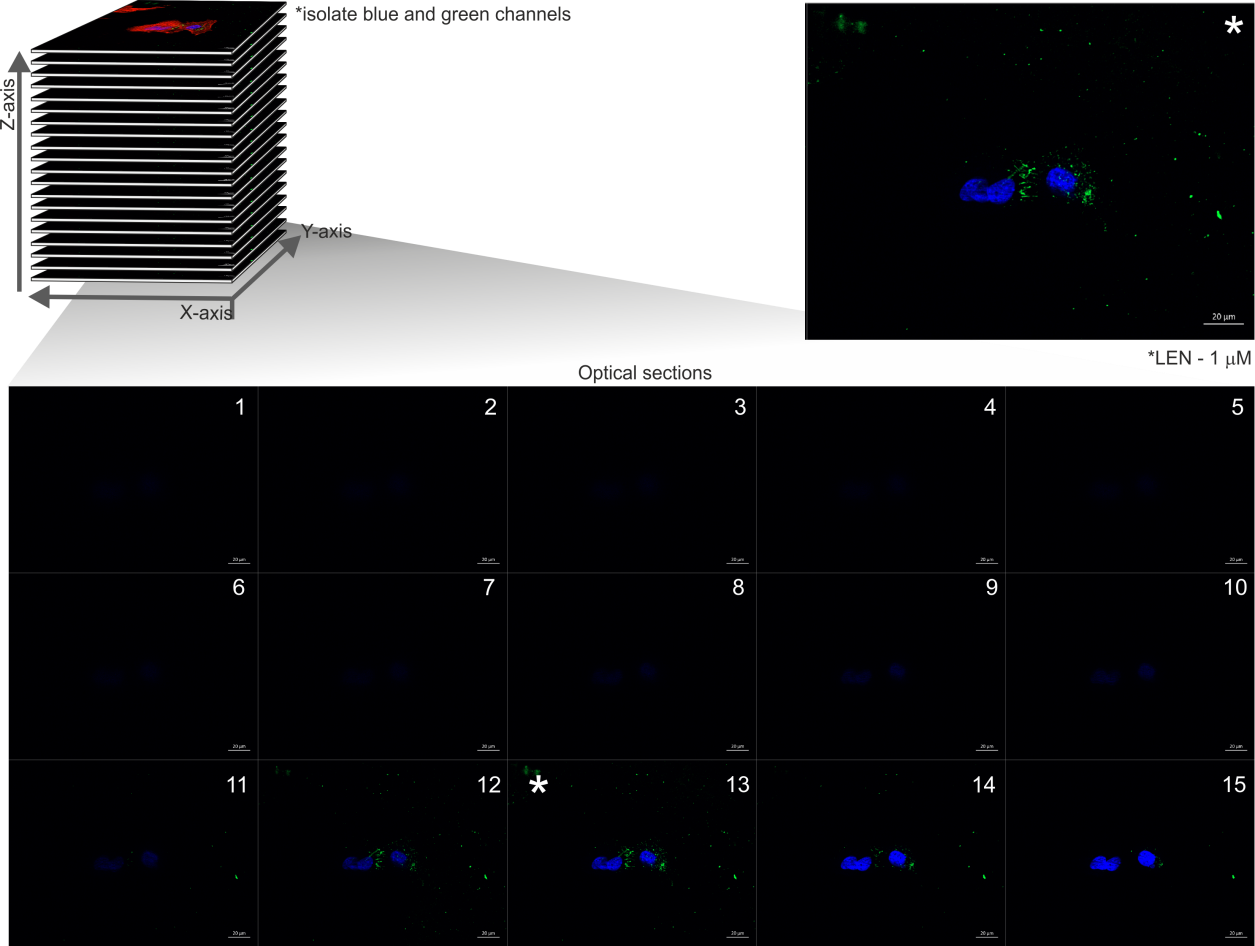

Supplement: S3 Fig — Optical sectioning of complete z-stacks reveals FITC-labelled LEN (green) is on the same focal plane as the cell nucleus (Hoechst–blue) indicating the VL is inside the cells and not surface bound. Top right panel shows enlarged image of z-slice 13 marked asterisks. (TIF) [file pone.0206167.s004.tif]

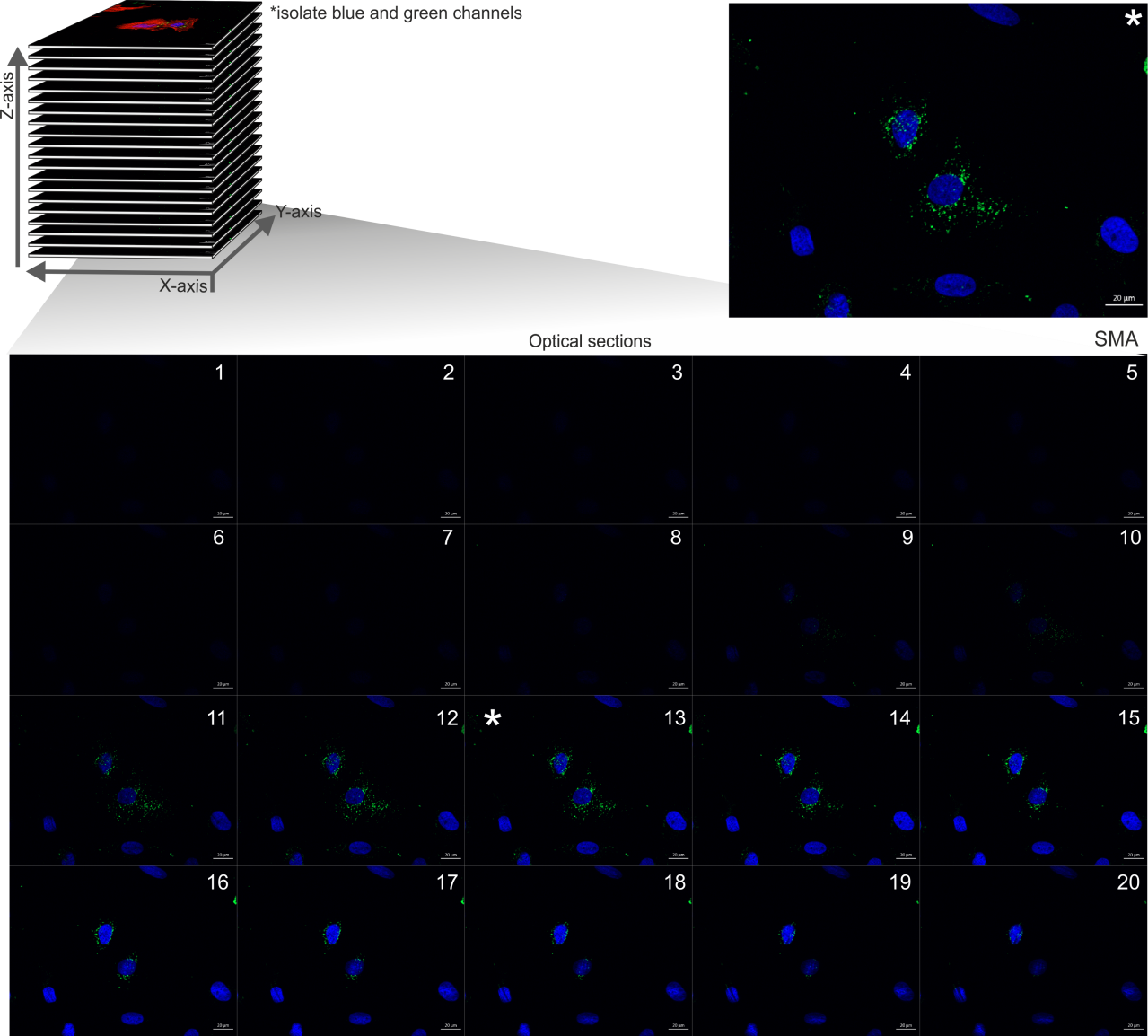

Supplement: S4 Fig — Optical sectioning of complete Z-stacks reveals FITC-labelled SMA (green) is on the same focal plane as the nuclei (Hoechst–blue) indicating the VL is inside the cells and not surface bound. Top right panel shows enlarged image of z-slice 13 marked asterisks. (TIF) [file pone.0206167.s005.tif]
